# Low disease risk and penetrance in Leber hereditary optic neuropathy

**DOI:** 10.1016/j.ajhg.2022.11.013

**Published:** 2022-12-23

**Authors:** Eloise C. Watson, Ryan L. Davis, Shyamsundar Ravishankar, Joseph Copty, Sarah Kummerfeld, Carolyn M. Sue

**Affiliations:** 1 Department of Neurogenetics, Kolling Institute, Faculty of Medicine and Health, University of Sydney and Northern Sydney Local Health District, Reserve Rd, St Leonards, NSW, Australia; 2 Department of Neurology, Royal North Shore Hospital, Northern Sydney Local Health District, St Leonards, NSW, Australia; 3 Department of Neurology, Wellington Hospital, Capital and Coast District Health Board, Newtown, Wellington, New Zealand; 4 Garvan Institute of Medical Research, Darlinghurst, NSW, Australia; 5 St Vincents Clinical School, UNSW, Sydney, NSW, Australia

**Keywords:** mitochondrial disease, Leber hereditary optic neuropathy, LHON, prevalence, penetrance, vision los, risk

## Abstract

The risk of Leber hereditary optic neuropathy (LHON) has largely been extrapolated from disease cohorts, which underestimate the population prevalence of pathogenic primary LHON variants as a result of incomplete disease penetrance. Understanding the true population prevalence of primary LHON variants, alongside the rate of clinical disease, provides a better understanding of disease risk and variant penetrance. We identified pathogenic primary LHON variants in whole-genome sequencing data of a well-characterized population-based control cohort and found that the prevalence is far greater than previously estimated, as it occurs in approximately 1 in 800 individuals. Accordingly, we were able to more accurately estimate population risk and disease penetrance in LHON variant carriers, validating our findings by using other large control datasets. These findings will inform accurate counseling in relation to the risk of vision loss in LHON variant carriers and disease manifestation in their family. This Matters Arising paper is in response to Lopez Sanchez et al. (2021), published in *The American Journal of Human Genetics*. See also the response by Mackey et al. (2022), published in this issue.

## Main text

We read with interest the article by Lopez Sanchez and colleagues, examining the epidemiology and risk of vision loss in Leber hereditary optic neuropathy (LHON) in Australia through review of a national clinical register.^1^ Based on this disease cohort, they estimated that 1 in 4,600 Australian individuals carry a primary LHON mtDNA mutation but only 1 in 68,403 individuals are clinically affected by LHON. This suggests a lower penetrance of disease among mutation carriers (17.5% for males and 5.4% for females; 12.5% overall) than previous reports and challenges the oft-quoted 50% risk of vision loss in males with a primary LHON mutation.^2^^,^^3^^,^^4^^,^^5^^,^^6^^,^^7^^,^^8^

Three primary mutations in *MT-ND1* (m.3460G>A [p.Ala52Thr]), *MT-ND4* (m.11778G>A [p.Arg340His]), and *MT-ND6* (m.14484T>C [p.Met64Val]) account for 90%–95% of clinical cases of LHON (mitochondrial nucleotide numbering is based on GenBank: NC_012920). The m.11778G>A mutation is the most common cause of LHON in Northern Europe, Australia, and the Far East,^9^^,^^10^^,^^11^^,^^12^ while the m.14484T>C mutation is the most common cause amongst French-Canadians, attributed to a founder effect.^13^^,^^14^^,^^15^ Although mutations are typically homoplasmic, disease penetrance is variable, influenced by mtDNA haplogroup, age, gender, and environmental factors.^6^^,^^16^^,^^17^^,^^18^^,^^19^ *De novo* mutations have been reported, but sporadic disease presentations have been attributed to limitations in tracing symptomatic family history.^20^

Consistent with data from Lopez Sanchez et al., the prevalence of clinically manifest LHON has been estimated at 1 in 60,000 in Australia on the basis of blindness registries.^21^ Internationally, estimates from geographically constrained Western European disease cohorts vary from 1 in 27,000–31,000 in the North East of England^22^^,^^23^^,^^24^ to 1 in 39,000 in the Netherlands,^8^ 1 in 50,000 in Finland,^6^ and 1 in 54,000 in Denmark.^25^

Population prevalence of the three primary LHON mutations has also been estimated from disease cohorts, ranging from 1 in 8,600 to 1 in 13,500,^6^^,^^22^^,^^23^^,^^24^^,^^26^ compared to 1 in 4,600 estimated by Lopez Sanchez et al.^1^ However, using a population-based approach, Elliot et al.^27^ found the prevalence of LHON mutations in the general population may be much higher, as 1 in 350 individuals (nine of 3,168 neonates tested; three homoplasmic) harbor a primary LHON variant, indicating a greater population at risk of developing LHON than disease cohort estimates imply.

Lopez Sanchez et al.^1^ emphasized the importance of accurate epidemiological data to inform genetic counseling for those with LHON and their family members. This is critical in an era of increasingly available genomic sequencing and a broader range of therapeutic options, and so it requires accurate estimates of both population prevalence of LHON mutations and the prevalence of clinically manifest disease. The majority of LHON epidemiological data, however, is extrapolated from disease cohorts and therefore subject to sampling bias. Large-scale population-based prevalence studies have been impeded by the cost of sequencing, the rarity of LHON, and the age of disease onset. However, community-based prevalence studies have been successful in accurately predicting the true prevalence of mtDNA mutations in the population.^28^^,^^29^ More recent development of large population-based genetic reference cohorts provides the opportunity for orthogonal estimates of LHON mutation prevalence, disease risk, and penetrance in the wider population.

Recognizing the potential clinical implications of the gap between population-based estimates of LHON mutation frequency and those derived from disease cohorts, such as in Lopez Sanchez et al., we sought to clarify population prevalence of primary LHON mutations in a “healthy” control cohort. Utilizing the Medical Genome Reference Bank,^30^ comprising whole-genome sequencing data from blood DNA of 4,012 healthy elderly Australians, we called mtDNA variants by using a mitochondrial variant analysis tool, *mity*.^31^ Homoplasmic (>95%) pathogenic LHON mutations were extracted and analyzed with custom scripts and R studio (version 4.0.5). Five individuals from three different haplogroups harbored primary LHON mtDNA mutations at homoplasmic levels; four had the m.14484T>C variant (haplogroups H2a2, H2a2a1, U5a2d1a, and U5b1g) and one the m.11778G>A variant (haplogoup X2p1; haplogroups determined with Haplogrep 2.1.21,^32^ with PhyloTree Build 17^33^). No individuals in this cohort harbored a homoplasmic m.3460G>A variant. On the basis of these data, as many as 125 per 100,000 individuals (95% CI 40–291), or 1 in 800 people, may carry a homoplasmic primary LHON mutation. Laricchia and colleagues identified similar numbers in the gnomAD (v.3.1) genome database: approximately 1 in 900 individuals carry one of the three primary LHON mutations (m.14484T>C, 1 in 1,526; m.11778G>A, 1 in 2,351; m.3460G>A, 1 in 56,426).^34^ Similarly, Bolze et al. found in their HelixMTdb dataset containing 195,983 mitochondrial genomes that 1 in 1,360 individuals carried a homoplasmic m.14484T>C mutation.^35^ It is interesting to note that the relative population frequencies of the three mutations, rather than reflecting their relative frequency in causing clinically manifest disease, inversely relate to the severity of the biochemical deficit they cause,^36^ highlighting the variable penetrance of the different mutations. Given the influence of mtDNA genetic background, as defined by haplogroup, on LHON mutation penetrance, it is also instructive to note the mtDNA genetic background on which these identified mutations have arisen. There is considerable evidence in European populations supporting increased penetrance of the m.14484T>C and, to a lesser extent, the m.11778G>A variant in association with haplogroup J, while haplogroup H appears protective.^17^^,^^37^ Interestingly, none of the four m.14484T>C mutations or the m.11778G>A mutation identified in the Medical Genome Reference Bank associated with haplogroup J, consistent with reduced disease penetrance in the absence of this genetic background,^38^ while haplogroup U is disproportionately represented amongst m.14484T>C carriers (2 of 4, 50%) compared to the proportion of haplogroup U individuals in the MGRB cohort (373 of 4,012, 9.3%). By contrast, the most biochemically severe m.3460G>A mutation, absent in this cohort, may not require a specific mtDNA genetic background to express LHON.

Assuming a high proportion of Australian LHON cases were identified by Lopez Sanchez et al., for an Australian population of 25,739,000 (source: Australian Bureau of Statistics, accessed January 17, 2022, web resources) with population mutation prevalence estimated from the MGRB cohort (5 in 4,012) and the number of live disease cases identified by Lopez Sanchez et al. (n = 355), the overall penetrance of the three primary LHON mutations is calculated to be 1.11% (95% CI 0.5%–3.4%):

NumberofreportedindividualswithLHONvisionlossinAustralia=355

$$Number\;of\;carriers\;of\;a\;pathogenic\;LHON\;mutation\;in\;Australia\;estimated\;from\;the\;MGRB=\left(\frac{5}{4012}\right)\times 25,739,000=32,078$$

$$Estimated\;penetrance\;of\;primary\;LHON\;mutations\;in\;Australia=\left(\frac{355}{32,078}\right)\times 100=1.11\%$$

Case identification by clinical and self-referral, as utilized by Lopez Sanchez et al., is subject to sampling bias and may underestimate the true prevalence of LHON. Even allowing for a 10-fold underestimation of LHON prevalence by this method, penetrance would still only be estimated at 11.1%. These data support the finding that the risk of vision loss in carriers of a primary LHON mutation may be significantly lower than cited and even lower than Lopez Sanchez and colleagues’ data indicates.^1^ This is particularly important for the m.14484T>C mutation, observed at higher frequency in population-based cohorts^27^^,^^34^^,^^35^ but lower frequency in individuals with LHON vision loss,^9^^,^^10^^,^^11^^,^^12^ suggesting additional genetic and/or environmental factors may modify risk.

Estimating carrier frequency from a “healthy” population cohort avoids the bias inherent in disease-based epidemiological studies. Although the MGRB is a relatively small cohort, identified mutation frequencies are consistent with the findings of Elliot et al.^27^ and those observed in larger international cohorts.^34^^,^^35^ In addition, the elderly nature of the MGRB cohort renders incident disease unlikely. However, the healthy status of the cohort may overestimate the risk of vision loss associated with LHON mutations if individuals with significant visual loss were excluded from recruitment.

Appropriate genetic counseling is critical for individuals and families with an LHON variant, especially as therapeutic and family planning options are of increasing relevance and insurance considerations can also be affected by the diagnosis and prognosis.^1^ Although Lopez Sanchez et al. identified that the risk of vision loss within a pedigree may best inform risk for individuals within that pedigree,^1^ the estimate presented here adds important information for the family members who are asymptomatic carriers. Furthermore, accessible data from population cohorts invites additional investigation and elucidation of potential genetic resilience and susceptibility factors that may influence disease manifestation in mutation carriers.

## Data Availability

The Medical Genome Reference Bank dataset analyzed in this study is available by application at https://sgc.garvan.org.au/initiatives/mgrb. Publicly available allele frequency datasets are accessible at https://gnomad.broadinstitute.org/ and at https://www.helix.com/pages/mitochondrial-variant-database. The tool *mity*, used for whole-genome sequencing analysis of mtDNA variants, is available at https://github.com/KCCG/mity.

## Web resources

Australian Bureau of Statistics, https://abs.gov.au/
